# GP awareness, practice, knowledge and confidence: evaluation of the first nation-wide dementia-focused continuing medical education program in Australia

**DOI:** 10.1186/s12875-020-01178-x

**Published:** 2020-06-10

**Authors:** Anne-Nicole Casey, M. Mofizul Islam, Heike Schütze, Anne Parkinson, Laurann Yen, Allan Shell, Margaret Winbolt, Henry Brodaty

**Affiliations:** 1 Dementia Centre for Research Collaboration, University of New South Wales (UNSW) Sydney, AGSM Building, Sydney, NSW 2052 Australia; 2 Centre for Healthy Brain Ageing, School of Psychiatry, UNSW Sydney, Sydney, NSW 2052 Australia; 3 Department of Public Health, School of Psychology and Public Health, La Trobe University, Melbourne, VIC 3086 Australia; 4 School of Health and Society, University of Wollongong, Wollongong, NSW 2522 Australia; 5 School of Public Health and Community Medicine, UNSW Sydney, Sydney, NSW 2052 Australia; 6 Department of Health Services Research & Policy, Research School of Population Health, Australian National University, Canberra, NSW 2601 Australia; 7 Dementia Training Australia, La Trobe University, Melbourne, VIC 3086 Australia; 8 Academic Department for Old Age Psychiatry, Prince of Wales Hospital, Randwick, NSW 2031 Australia

**Keywords:** Alzheimer’s disease, Dementia, Dementia care, Applied knowledge translation, Timely diagnosis, Continuing professional development, General practice

## Abstract

**Background:**

Dementia is under-diagnosed in primary care. Timely diagnosis and care management improve outcomes for patients and caregivers. This research evaluated the effectiveness of a nationwide Continuing Medical Education (CME) program to enhance dementia-related awareness, practice, knowledge and confidence of general practitioners (GPs) in Australia.

**Methods:**

Data were collected from self-report surveys by GPs who participated in an accredited CME program face-to-face or online; program evaluations from GPs; and process evaluations from workshop facilitators. CME participants completed surveys at one or more time-points (pre-, post-program, six to 9 months follow-up) between 2015 and 2017. Paired samples t-test was used to determine difference in mean outcome scores (self-reported change in awareness, knowledge, confidence, practice) between time-points. Multivariable regression analyses were used to investigate associations between respondent characteristics and key variables. Qualitative feedback was analysed thematically.

**Results:**

Of 1352 GPs who completed a survey at one or more time-points (pre: 1303; post: 1017; follow-up: 138), mean scores increased between pre-CME and post-program for awareness (*M*_*post-pre*_ = 0.9, *p* <  0.0005), practice-related items (*M*_*post-pre*_ = 1.3, *p* <  0.0005), knowledge (*M*_*post-pre*_ = 2.2, *p* <  0.0005), confidence (*M*_*post-pre*_ = 2.1, *p* <  0.0005). Significant increases were seen in all four outcomes for GPs who completed these surveys at both pre- and follow-up time-points. Male participants and those who had practised for five or more years showed greater change in knowledge and confidence. Age, years in practice, and education delivery method significantly predicted post-program knowledge and confidence. Most respondents who completed additional program evaluations (> 90%) rated the training as relevant to their practice. These participants, and facilitators who completed process evaluations, suggested adding more content addressing patient capacity and legal issues, locality-specific specialist and support services, case studies and videos to illustrate concepts.

**Conclusions:**

The sustainability of change in key elements relating to health professionals’ dementia awareness, knowledge and confidence indicated that dementia CME programs may contribute to improving capacity to provide timely dementia diagnosis and management in general practice. Low follow-up response rates warrant cautious interpretation of results. Dementia CME should be adopted in other contexts and updated as more research becomes available.

## Background

Dementia (Major Neurocognitive Disorder) is a syndrome characterised by deterioration in memory, problem-solving and behaviour resulting in reduced functional ability in daily activities [1]. Dementia is typically progressive and irreversible. It may result from a number of diseases such as Alzheimer’s and cerebrovascular disease [1]. It is the second leading cause of death in Australia [2] and the fifth leading cause of death globally [3]. Dementia is a worldwide public health priority [4, 5]; an estimated 47 million people have dementia and 9.9 million new cases are diagnosed each year [6]. Despite growing insight into the global burden of dementia, healthcare practitioners’ attitudes are not keeping pace with evidence [7]. A recent global survey of over 14,000 healthcare practitioners indicated that 62% still believe that dementia is a normal part of ageing [7]. Greater translation and delivery of dementia-related knowledge into practice are needed.

General practitioners (GPs) are the customary first point of contact for people with dementia and their caregivers. They play a pivotal role in symptom recognition, assessment and referral [8] and are ideally placed to co-ordinate continuing care and support [9, 10]. Dementia diagnosis rates in primary care are suboptimal [11–13] and the need to improve the approach to dementia in primary care is well-acknowledged [11–18]. Under-detection may contribute to poorer outcomes through lost opportunity to address reversible causes [8], belated symptomatic treatment [19], and inadequate provision of support [20]. People from racially and culturally diverse backgrounds [21] experience longer delay to specialist consultation after initial diagnosis and may have poorer outcomes [22]. Similarly, risk of poorer outcomes may be amplified for people with younger-onset dementia who experience accelerated disease progression and paradoxically commonly encounter delay in appropriate intervention as a result of misdiagnosis [23]. Timely detection increases therapeutic options for practitioners and consumers [19, 24], and enables more potentially effective care management [9, 10]. Therapeutic windows exist during which time pharmaceutical and non-pharmaceutical therapies [8, 20], such as goal-oriented cognitive rehabilitation [24], may be introduced to help some people with mild to moderate dementia to improve their daily functioning and meet individual goals [25, 26].

The timely diagnosis of dementia in primary care requires GPs to be able to differentiate normal ageing from dementia [27, 28]. Differential diagnosis entails familiarity with time-efficient screening tools and confidence in assessment [29–31]. Efficient management of dementia care involves knowledge of treatment availability and community consumer-support resources [32–34]. GPs commonly report that they receive insufficient pre- and post-qualification training in dementia [14, 33, 35–38]. Practitioner age and experience level influence attitudes, awareness, level of confidence and practice intention regarding the timely diagnosis and management of dementia [35, 39, 40]. Given the reported limitations of dementia-related medical training for students and registrars [35, 41], and the essential role of GPs in dementia diagnosis and care particularly for people who may not use specialist services [22], there is urgent need for dementia-related professional development for GPs.

Continuing Medical Education (CME) are ongoing education and retraining programs for licensed medical professionals incorporating clinical guidelines and best research evidence to foster demonstrated changes in the performance, knowledge, skills, actions and attitudes of practicing professionals [42, 43]. CME dementia-focused education programs can be delivered face-to-face and online to reach large numbers of GPs [44, 45]. Individual characteristics such as age and geographic location may influence whether health professionals choose face-to-face or online formats [44, 46–48]. GPs who participate in dementia-focused CME programs self-report significantly increased knowledge and confidence in their dementia care competency from pre- to post-education [43, 49, 50]. However, evidence for longitudinal effects of educational interventions to promote timely diagnosis in general practice is equivocal [28, 51, 52].

The Supporting GPs and Practice Nurses in the Timely Diagnosis of Dementia project was commissioned by Dementia Australia (formerly Alzheimer’s Australia) [53]. The project updated and expanded the delivery and evaluation of the first nationwide program in Australia to improve GP assessment, diagnosis and management of dementia [45, 53] through providing an accredited Royal Australian College of General Practitioners (RACGP) and Australian College of Rural and Remote Medicine (ACRRM) CME program. Whilst the CME program was designed for GPs, enrolment was also open to primary health care nurses and other health care professionals practising in major cities and in metro and regional areas [45, 53]. This article focuses on outcomes for the GPs who participated in the program from 2015 to 2017 [53].

In accordance with RACGP guidelines, the CME program comprised a minimum of 6 hours of thematically-linked structured educational content, including at least two-thirds interactive or experiential content (such as case studies and discussion) and three to five learning outcomes [42, 45]. The program’s educational content was based largely on Brodaty et al. (2013a) “*Dementia: 14 Essentials of assessment and care planning”* [9] and (2013b) “*Dementia: 14 Essentials of management*” [10]. Development of the program’s educational content has been described in detail elsewhere [45, 53, 54].

In order to obtain CME points, GPs were required to engage with the educational content and to complete three additional activities including a predisposing activity, a reinforcing activity and a program evaluation feedback form [42]. The CME program was offered in three formats: online modules, large group face-to-face workshops in major cities at the General Practice Conference and Exhibition (GPCE), and small group face-to-face workshops in metropolitan and regional areas. The online format comprised six 60-min modules. The face-to-face formats comprised four 90-min sessions: delivered across 2 days in GPCE large group workshops and delivered in 1 day at small group workshops [53]. GPs obtained 40 CME points for completing all 6 hours of face-to-face or online education and the three required activities [42]. An introductory 90-min overview was also offered at GPCE to obtain three CME points [53]. Online participants could choose to take one or more individual modules for two CME points per module. GP registrars, medical graduates undertaking advanced training in general practice, were offered a 2-h face-to-face workshop toward fulfilment of training curriculum requirements.

The aim of this research was to evaluate the CME program’s effectiveness in terms of GP self-rated awareness, practice, knowledge and confidence, immediately upon completion of the CME program and again six to nine months later.

## Methods

### GP recruitment and participants

The CME training in dementia care was advertised along with other CME courses available to health care professionals, including GPs [53, 55]. Participants in this study were a convenience sample of GPs, GP registrars and international medical graduates in general practice settings who enrolled for CME training in dementia care, as well as GPs and geriatricians who facilitated the face-to-face workshops. Those who engaged in the CME were provided an information sheet explaining the aims and focus of the research and that participation was voluntary. Return of the self-report survey indicated consent. Participants were requested to provide their contact email address for a six to nine month follow-up survey. Ethics approval for the study was obtained from the Human Research Ethics Committees of the Australian National University (HREC 2015/352), and the University of New South Wales Sydney, Australia (HC13019).

### Data collection

#### Self-report surveys

GPs completed a self-report survey immediately prior to commencement of the CME program, immediately after completion and six to nine months after completion of the program. Pre-CME and post-program survey data were collected between October 2015 and July 2017. Follow-up survey data were collected from February 2016 to April 2017. Funding constraints precluded further data collection.

The survey was developed in alignment with the accredited CME program and demographic questions were included. Author AS, a GP, checked the survey for readability as well as alignment with the topics taught in the CME program [54]. The face-to-face surveys were created first. These surveys were then adapted to fit an online template. The questions remained the same as the hard copy versions. The visual appearance of the online survey was adapted to fit the form and functionality of different electronic devices (computer, tablet, smartphone). All surveys were pilot tested with a small group comprising four academic colleagues and two GPs to determine anomalies and to confirm that the surveys were user friendly [53]. In accordance with feedback provided by authors AS and HB (a practising psychogeriatrician), minor changes were made to item wording regarding phrasing in items referencing use of psychotropic medication [53].

All survey items are noted in Table 2. The self-report surveys asked participants to rate their awareness (15 items) and current practice (8 items), each relating to a topic that had been covered in the CME program. Awareness was rated on a 4-point scale from “disagree strongly” to “agree strongly” (scores: − 2, − 1, 1, 2). Current practice was rated using a 5-point scale of “never 0%” to “always 100%” (scores: 0 to 4) [45].

Respondents ranked their knowledge level in dementia-focused care by choosing a number on an 11-point scale ranging from 0 “no knowledge” to 10 “expert”, and ranked how confident they were in dementia-focused care using a similar 11-point scale ranging from 0 “not at all” to 10 “completely” [53].

Respondents were asked demographic questions including age, sex, working status, professional role, practice location postcode, number of years in practice and practice profile. Practice location was coded using Accessibility and Remoteness Index of Australia (ARIA +) categories (Major Cities, Inner Regional, Outer Regional, Remote, Very Remote) [53].

Participants who attended face-to-face sessions completed paper-based pre- and post-program surveys. Participants who engaged with the education online began the CME with the pre-program survey and exited the CME with the post-program survey. All participants who provided an email address for follow-up received: 1) an emailed invitation to take part in the follow-up survey containing a link to the survey on SurveyMonkey; 2) an emailed reminder after 7 days; 3) an emailed reminder after 14 days.

#### Program evaluation

Program evaluation feedback forms required for CME point allocation were collected online and by the event organizer at GPCE events; research team members collected required forms from GPs attending small group workshops. GPs evaluated the CME program by rating the extent to which their learning needs were met (not met, partially, entirely) and the extent to which the program was relevant to their practice (not relevant, partially, entirely). GPs were also given the option to provide summative feedback about the program and recommendations for improving the program.

#### Process evaluation

All process evaluation data was collected between June and July 2017. Workshop facilitators received an emailed invitation to take part in the process evaluation. A copy of the questionnaire was attached to the emailed invitations. Facilitators were offered the choice of responding by completing and returning the questionnaire by email or by answering the questions over the phone with a research assistant. Workshop facilitators evaluated the program implementation process by answering open-ended questions regarding barriers and enablers to workshop delivery, aspects of the program format and workshop organization, and how to improve future workshop delivery. Questionnaire items are noted in Additional File 9: Table S8. All data were de-identified prior to analysis.

### Analysis

Survey response rates were calculated as the number of returned questionnaires divided by the total number of individuals who enrolled in the CME program. Descriptive statistics were used to analyse participant demographic characteristics. Due to small numbers in Outer Regional and Remote and Very Remote categories, Inner and Outer Regional were combined and recoded as “Regional” and Very Remote was coded with “Remote” for analyses. Chi-squared tests were used to compare characteristics of participating GPs with characteristics of GPs in Australia generally [56], and to compare characteristics of GPs who submitted surveys at all three time-points with GPs who submitted surveys only at pre-, or at pre- and post- CME program.

A composite index was calculated to represent participant level of awareness and current practice in managing care at each time point. Each composite index was based on a participant’s average response to survey items within each of the two categories (awareness and practice) and adjusted for missing values by dividing the total score by the total number of responses. Additionally, an overall average score was calculated for each item for awareness and for current practice. Mean scores were also calculated for knowledge and confidence, respectively, at each time point.

Immediate training effects were calculated for individual items for all respondents using the average difference between pre- and post- CME program scores. Subgroup analysis was used to explore potential variability in the effects of face-to-face and online delivery methods. Paired t-tests were used to compare the mean scores of six to nine month follow-up and pre-CME program from the subset of participants who completed surveys at all three time points in order to examine longitudinal effects of the program on the GPs’ overall awareness, practice, confidence and knowledge, respectively.

Pre- CME program scores were dichotomised for awareness (mean ≤ 0; mean > 0) and practice (mean ≤ 2; mean > 2). Logistic regression was performed controlling for participant characteristics (sex, number of years in practice, age, practice location) and program delivery method (online and face-to-face) to assess the factors significantly associated with: 1) the likelihood that GPs would endorse that they were aware, as opposed to unaware, of dementia diagnosis and management topics, and; 2) the likelihood that GPs would endorse that they applied recommended diagnosis and management actions in practice more than half the time, as opposed to less than half the time, prior to engaging in the CME program. Linear regression was performed, and the same variables were controlled in order to assess the impact of GP characteristics on knowledge and confidence at pre- and post-program.

The analysis of qualitative data synthesised and summarised both GP and facilitator comments and recommendations for improving the CME program. Examples of both GP and facilitator feedback were coded thematically, and results summarised in a narrative manner.

## Results

**Fig. 1:**
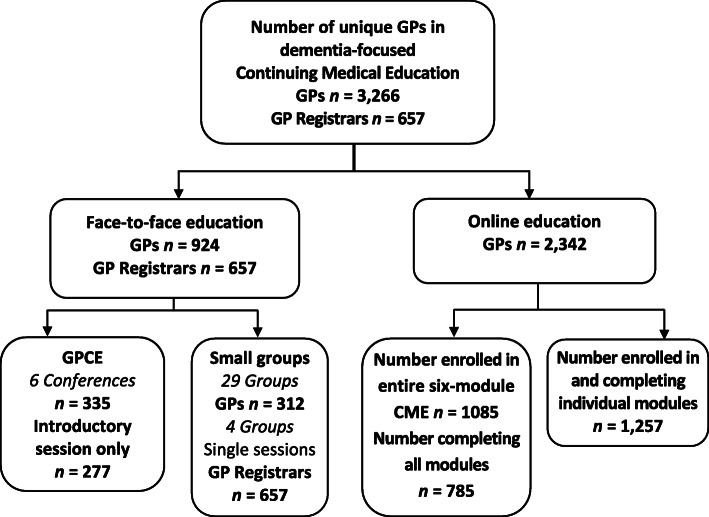
General Practitioner dementia-focused Continuing Medical Education uptake June 2015 through July 2017

Of 3923 unique GPs who participated in the educational program between July 2015 and July 2017 (Fig. 1), nearly 60% (*n* = 2342) engaged online rather than face-to-face. Of the 44% of GPs (*n* = 1732) who enrolled for the full 40 CME point program, 83% completed all required activities (face-to-face, 647; online, 785).

### Survey participant characteristics

**Fig. 2:**
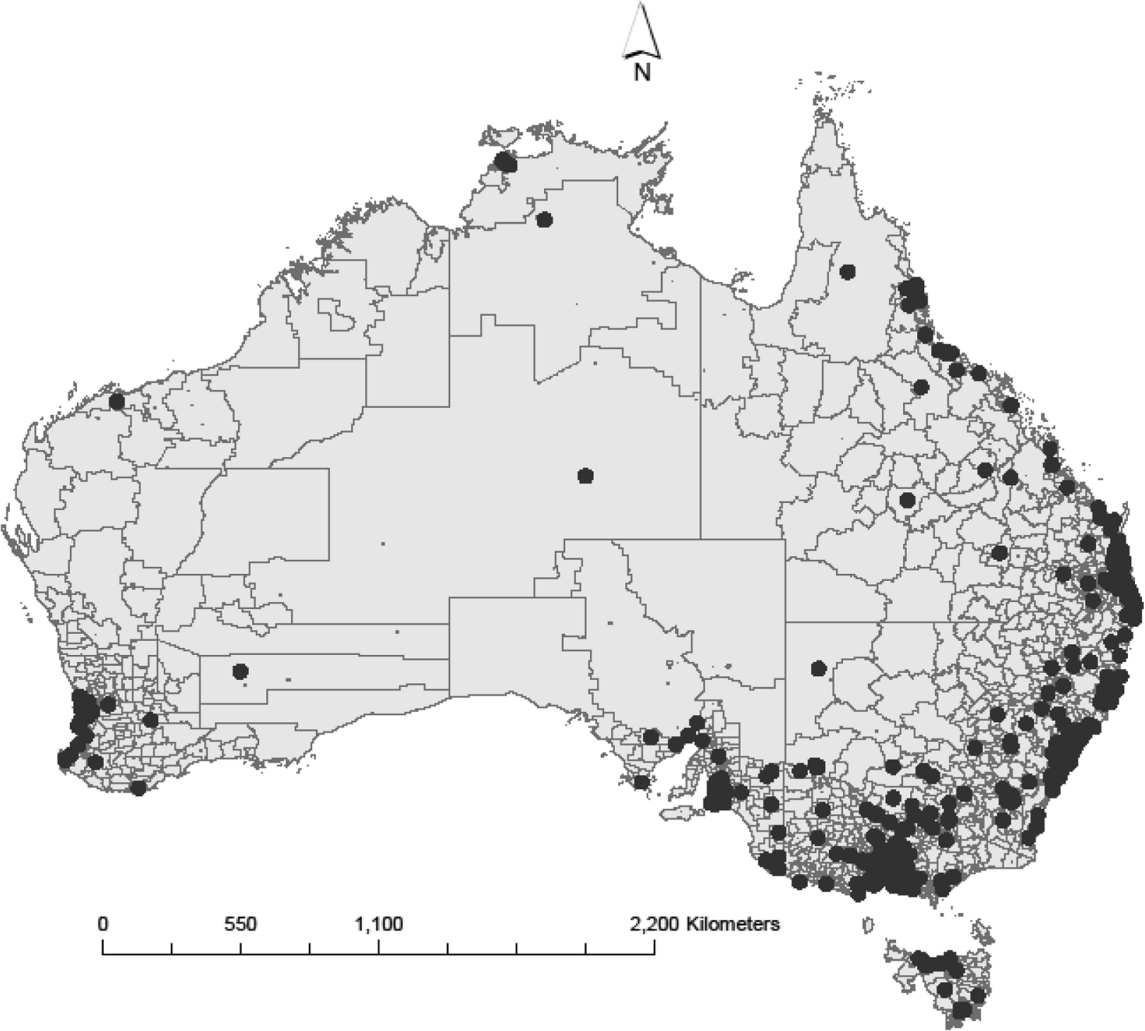
Practice location of participating General Practitioners who engaged in the dementia-focused Continuing Medical Education program. Map created using ArcGIS software (Environmental Systems Research Institute, Inc.1995–2020. https://www.esri.com/en-us/arcgis/about-arcgis/overview)

Of the unique participants (*N* = 3923), just over 33% (*n* = 1303) responded at baseline, 51.4% (*n* = 2017) responded at post-program, and 3.5% (*n* = 138) responded at follow-up (see Additional File 1: Fig. S1). The geographic distribution of survey participants’ practice postcodes is displayed in Fig. 2.

Nearly half (*n* = 653) of GPs who responded at baseline were 45 years of age or younger, 51.2% (*n* = 670) were women, 68.6% (*n* = 882) practised full-time and 19.2% (*n* = 259) were GP registrars. Over 36% (*n* = 451) practised in regional and remote areas. The majority of GP respondents (53.4%, *n* = 722) attended the program face-to-face (Table 1).

**Table 1:** Characteristics of General Practitioners who participated in Continuing Medical Education program evaluation surveys

	Program delivery method			
	Face-to-face		Online	
	Large group	Small group^a^		Total
Characteristics	(*n*, %)	(*n*, %)	(*n*, %)	(*n*, %)
Age in years (*n* = 1311)				
< 35	23, 8.7	207, 46.5	96, 15.9	326, 24.9
35 to 44	45, 17.0	91, 20.4	191, 31.7	327, 24.9
45 to 54	64, 24.2	59, 13.3	149, 24.8	272, 20.7
55 to 64	65, 24.6	50, 11.2	93, 15.4	208, 15.9
65+	67, 25.4	38, 8.5	73, 12.1	178, 13.6
Sex (*n* = 1309)				
Female	127, 48.1	267, 60.3	276, 45.8	670, 51.2
Male	137, 51.9	176, 39.7	326, 54.2	639, 48.8
Working status (*n* = 1286)				
Working full-time	149, 60.1	335, 76.8	398, 66.1	882, 68.6
Working part-time	99, 39.9	101, 23.2	204, 33.9	404, 31.4
Years in practice (*n* = 1326)				
< 5	25, 9.6	210, 47.7	143, 22.8	378, 28.5
5 to 10	28, 10.8	90, 20.5	152, 24.3	270, 20.4
11 to 15	29, 11.2	21, 4.8	87, 13.9	137, 10.3
16 to 20	26, 10.0	25, 5.7	59, 9.4	110, 8.3
> 20	152, 58.5	94, 21.4	185, 29.6	431, 32.5
Practice profile (*n* = 1262)				
Solo GP^b^	28, 11.4	39, 9.4	84, 14.0	151, 12.0
2 to 5 GPs	101, 41.2	141, 34.0	302, 50.2	544, 43.1
6+ GPs	116, 47.3	235, 56.6	216, 35.9	567, 44.9
Rurality^c^ (*n* = 1251)				
Major city	150, 62.8	215, 51.3	435, 73.4	800, 63.9
Regional	87, 36.4	198, 47.3	151, 25.5	436, 35
Remote	2, 0.8	6, 1.4	7, 1.2	15, 1.1

^a^ Small group participants included 258 registrars

^b^*GP* General Practitioner

^c^ “Rurality” based on Accessibility / Remoteness Index of Australia (ARIA+) remoteness ratings

A higher proportion of GP participants were female (45.4%, *p* <  0.0005) and less than 35 years of age (24.9%, *p* <  0.0005) compared to that reported nationally (see Additional File 2: Table S1) [56]. There was a significantly higher proportion of GPs working in regional areas (35%, *p* <  0.0005), and lower proportions of GPs working in major cities (63.9%, *p* = 0.001) and remote areas (1.1%, *p* <  0.0005), compared to the national workforce.

### Participant characteristics and choice of CME delivery method

Chi-square tests indicated an association between sex and choice of program delivery method *x*^2^ (1, *n* = 1309) = 13.48, *p* < 0.0005, with a higher proportion of males engaging online (50.9%, *n* = 325) compared with females (40.7%, *n* = 273). Practice location was associated with choice of program delivery method *x*^2^ (2, *n* = 1251) = 44.12, *p* < 0.0005. Compared to GPs practising in regional and remote areas, those practising in major cities were more likely to engage with the program online than face-to-face (see Additional file 3: Table S2).

### Short-term effects of the CME program

Average scores for the full sample for each awareness and practice item, respectively, at each of the three time-points are listed in Table 2. Scores in all key areas of self-reported awareness increased at post-program and follow-up when compared to pre- CME program, with slight declines between post-program and follow-up. The three topics with the lowest pre- CME program awareness scores, and subsequently also the three with the greatest increase between pre- and post- CME program were: the difference between mild cognitive impairment (MCI) and dementia, legal issues and dementia, and the cumulative anticholinergic effect of medications which can negatively affect cognition.

**Table 2:** Mean pre-, post-, six to nine month follow-up scores of GP awareness, practice, knowledge, confidence

	Pre-CME^a^ program score, Mean (*SD*)^b^			Post-program score, Mean (*SD*)			Six to nine month follow-up score, Mean (*SD*)		
	Face-to-face	Online	Total	Face-to-face	Online	Total	Face-to-face	Online	Total
Awareness topics (range − 2 to 2)^c, d^	(*n* = 698–711)	(*n* = 591)	(*n* = 1289–1302)	(*n* = 652–661)	(*n* = 356)	(*n* = 1008–1017)	(*n* = 86–88)	(*n* = 50–51)	(*n* = 136–139)
1.1 Early warning signs for dementia	0.7 (0.8)	0.9 (0.8)	0.8 (0.8)	1.5 (0.5)	1.5 (0.6)	1.5 (0.6)	1.3 (0.5)	1.4 (0.5)	1.3 (0.5)
1.2 Different types of dementia	0.8 (0.8)	0.9 (0.9)	0.8 (0.8)	1.5 (0.5)	1.6 (0.6)	1.5 (0.6)	1.2 (0.6)	1.3 (0.6)	1.2 (0.6)
1.3 Difference between Mild Cognitive Impairment (MCI) and dementia	−0.3 (1.1)	0.5 (1.1)	0.1 (1.2)	1.5 (0.6)	1.5 (0.6)	1.5 (0.6)	0.8 (1.0)	1.1 (0.9)	0.9 (0.9)
1.4 Barriers to diagnosis	0.4 (1.0)	0.8 (0.9)	0.6 (1.0)	1.4 (0.6)	1.4 (0.6)	1.4 (0.6)	1.2 (0.6)	1.3 (0.6)	1.2 (0.6)
1.5 Consequences of not recognizing dementia	0.6 (1.0)	1.0 (0.8)	0.8 (0.9)	1.4 (0.6)	1.5 (0.7)	1.4 (0.6)	1.4 (0.6)	1.5 (0.6)	1.4 (0.6)
1.6 Impact of co-morbidities on dementia diagnosis	0.5 (1.0)	1.0 (0.9)	0.7 (1.0)	1.4 (0.6)	1.5 (0.6)	1.4 (0.6)	1.4 (0.6)	1.4 (0.6)	1.4 (0.6)
1.7 Behavioral and psychological symptoms of dementia (BPSD)	0.6 (0.9)	0.9 (0.9)	0.7 (0.9)	1.5 (0.6)	1.5 (0.6)	1.5 (0.6)	1.3 (0.5)	1.4 (0.5)	1.3 (0.5)
1.8 Caregiver management education, support and referral	0.01 (1.1)	0.8 (1.0)	0.4 (1.1)	1.3 (0.7)	1.5 (0.6)	1.4 (0.7)	1.2 (0.7)	1.2 (0.7)	1.2 (0.7)
1.9 Legal issues and dementia	−0.01 (1.1)	0.8 (1.0)	0.3 (1.1)	1.3 (0.6)	1.5 (0.6)	1.4 (0.6)	1.0 (0.7)	1.2 (0.4)	1.1 (0.6)
1.10 Non-pharmacological management methods for BPSD	0.1 (1.1)	0.7 (1.0)	0.4 (1.1)	1.3 (0.7)	1.4 (0.6)	1.4 (0.7)	1.1 (0.7)	1.3 (0.6)	1.2 (0.6)
1.11 Pharmacological management methods for BPSD	0.1 (1.1)	0.7 (1.0)	0.4 (1.1)	1.3 (0.7)	1.4 (0.7)	1.4 (0.7)	1.1 (0.6)	1.3 (0.6)	1.1 (0.6)
1.12 Use of acetylcholinesterase inhibitors (AChEI) for the management of patients with dementia	0.3 (1.0)	0.7 (1.0)	0.5 (1.0)	1.3 (0.6)	1.4 (0.7)	1.4 (0.6)	1.1 (0.5)	1.0 (0.7)	1.1 (0.6)
1.13 The cumulative anticholinergic effect of medications which can negatively affect cognition in all people especially the elderly	−0.01 (1.7)	0.7 (1.0)	0.3 (1.4)	1.4 (0.7)	1.4 (0.7)	1.4 (0.7)	1.3 (0.6)	1.1 (0.7)	1.2 (0.7)
1.14 The fact that people with dementia are particularly sensitive to adverse effects of medications on cognition such as anticholinergics and sedatives	0.4 (1.1)	0.9 (0.9)	0.6 (1.0)	1.5 (0.6)	1.5 (0.6)	1.5 (0.6)	1.5 (0.6)	1.2 (0.7)	1.4 (0.6)
1.15 The importance of diagnosis and management of dementia in my practice	0.9 (0.9)	1.1 (0.9)	1.0 (0.9)	1.5 (0.5)	1.6 (0.6)	1.6 (0.6)	1.7 (0.5)	1.4 (0.6)	1.6 (0.5)
	Face-to-face	Online	Total	Face-to-face	Online	Total	Face-to-face	Online	Total
Practice topics (range 0–4)^e, f^	(*n* = 693–703)	(*n* = 591)	(*n* = 1284–1294)	(*n* = 650–658)	(*n* = 356)	(*n* = 1006–1014)	(*n* = 86–87)	(*n* = 47–49)	(*n* = 134–136)
2.1 Use assessment tools to help diagnose dementia	2.3 (1.2)	2.4 (1.1)	2.4 (1.1)	3.5 (0.6)	3.4 (0.7)	3.5 (0.7)	3.1 (0.7)	3.1 (0.8)	3.1 (0.8)
2.2 Use pharmacological management methods for BPSD	1.5 (1.0)	1.8 (1.0)	1.6 (1.0)	2.5 (1.0)	2.8 (0.9)	2.6 (1.0)	2.0 (0.8)	2.3 (0.9)	2.1 (0.8)
2.3 Use non-pharmacological management methods for BPSD	1.9 (1.1)	2.1 (1.0)	2.0 (1.0)	3.4 (0.7)	3.3 (0.7)	3.4 (0.7)	2.8 (0.8)	2.8 (0.8)	2.8 (0.8)
2.4 Refer patients to Alzheimer‘s Australia^g^	1.5 (1.2)	2.0 (1.2)	1.7 (1.2)	3.4 (0.8)	3.4 (0.8)	3.4 (0.8)	2.5 (1.1)	2.7 (1.1)	2.6 (1.1)
2.5 Assess caregiver stress	2.4 (1.0)	2.5 (1.0)	2.4 (1.0)	3.6 (0.6)	3.5 (0.7)	3.5 (0.6)	3.2 (0.8)	3.2 (0.8)	3.2 (0.8)
2.6 Refer caregivers for counselling	1.8 (1.0)	2.1 (1.1)	1.9 (1.1)	3.2 (0.8)	3.3 (0.8)	3.2 (0.8)	2.4 (0.9)	2.4 (1.2)	2.4 (1.0)
2.7 Counsel patients and their families about legal issues	1.8 (1.1)	2.3 (1.1)	2.0 (1.1)	3.5 (0.7)	3.5 (0.7)	3.5 (0.7)	2.8 (0.9)	1.0 (1.0)	2.8 (1.0)
2.8 Consider the safety of my patient with dementia to drive	2.8 (1.1)	1.0 (1.0)	2.9 (1.0)	3.7 (0.5)	3.6 (0.6)	3.7 (0.6)	3.6 (0.6)	3.6 (0.7)	3.6 (0.6)
	Face-to-face	Online	Total	Face-to-face	Online	Total	Face-to-face	Online	Total
Self-perceived level of Knowledge and Confidence (range 0–10)^h^	(*n* = 711)	(*n* = 591)	(*n* = 1302)	(*n* = 660)	(*n* = 356)	(*n* = 1016)	(*n* = 88)	(*n* = 49)	(*n* = 137)
Knowledge	5.0 (1.6)	5.2 (2.0)	5.1 (1.8)	7.1 (1.2)	7.6 (1.1)	7.3 (1.2)	7.0 (1.1)	7.3 (1.2)	7.1 (1.1)
Confidence	5.1 (1.8)^i^	5.2 (1.9)	5.1 (1.9)^j^	7.1 (1.3)	7.7 (1.2)	7.3 (1.3)	7.3 (1.3)	7.6 (1.2)	7.4 (1.3)

^a^CME, Continuing Medical Education

^b^*SD*, Standard deviation

^c^Awareness pre-education question-prompt “Prior to attending this workshop I was aware of …”; Post-education question-prompt “Now that I have completed this training I am aware of …”; and Follow-up question-prompt “Currently I am aware of …”

^d^Value index for awareness-related items: Disagree strongly = − 2, Disagree = − 1, Agree = 1, Agree strongly = 2

^e^Practice Pre-education question-prompt “I currently …”; Post-education question-prompt “Now that I have completed this training I plan to …”; and Follow-up question-prompt “In my practice currently I …”

^f^Value for index for practice-related items: Never = 0, Rarely = 1, Half the time = 2, Usually = 3, Always = 4

^g^Now *Dementia Australia*

^h^Likert scale for Knowledge and Confidence: 0–10

^i^*n* = 710

^j^*n* = 1301

Similarly, scores in all key areas of current practice increased at post-program and follow-up when compared to pre- CME program, with slight declines between post-program and follow-up (Table 2). The three topics with the lowest pre- CME program practice scores, and subsequently also the three with the greatest increase between pre- and post-program were: the use of non-pharmacological management methods for Behavioural and Psychological Symptoms of Dementia (BPSD), referring patients to Alzheimer’s Australia (now named Dementia Australia), and counselling patients and their families about legal issues.

Scores increased at post-program and follow-up in both self-reported knowledge and confidence when compared to pre- CME program scores (Table 2). Results indicated minor reductions in six to nine month follow-up knowledge scores when compared with post-program knowledge.

Results of analyses using scores for the subset of participants who completed both pre- and post-program surveys indicated that scores increased between pre- and post-program for GP awareness [*M*_post-pre_ = 0.9, *t*_911_ = 38.18, *p* < 0.0005], practice (*M*_post-pre_ = 1.3 *t*_908_ = 48.00, *p* < 0.0005), knowledge (*M*_post-pre_ = 2.2, *t*_910_ = 43.50, *p* < 0.0005), and confidence (*M*_post-pre_ = 2.1, *t*_910_ = 42.54, *p* < 0.0005). Sensitivity analyses indicated that registrars had slightly lower scores compared to GPs pre- CME program; scores increased significantly for both registrars and GPs between pre- and post-program (see Additional file 4: Table S3).

### Longer-term effects of the CME program

GPs who submitted surveys at all three time-points were significantly different to GPs who submitted surveys only at pre- CME program, or at pre- and post-program. There were no registrars in this subset. Nearly half (47.8%, *n* = 45) of the GP respondents were 55 years of age or older, over half (57%, *n* = 53) had been in practice more than 20 years, and nearly 90% had engaged face-to-face. These GPs reported higher mean scores at six to nine months follow-up than reported pre- CME program in all four outcomes (Table 3). Scores indicated only minor decreases from post-program to follow-up in all outcomes except self-reported confidence.

**Table 3:** Changes in mean scores for GPs who submitted surveys at each of three time-points

Questions	Pre-CME^a^ program	Post-program	Follow-up	Difference between Pre-CME program and Follow-up	Test statistic	95% Confidence Interval	*p*^c^ (two-sided)
	Mean (*SD*)^b^	Mean (*SD*)	Mean (*SD*)	Mean (*SD*)			
Awareness^d^	0.5 (0.6)	1.5 (0.4)	1.2 (0.4)	0.8 (0.7)	*t*_94_ = 10.00	0.61, 0.92	< 0.0005
Practice^e^	2.2 (0.6)	3.3 (0.5)	2.8 (0.5)	0.6 (0.6)	*t*_91_ = 9.00	0.44, 0.70	< 0.0005
Knowledge^f^	5.4 (1.8)	7.3 (7.3)	7.1 (1.1)	1.7 (1.6)	*t*_93_ = 9.79	1.32, 2.00	< 0.0005
Confidence^f^	5.4 (1.9)	7.4 (1.2)	7.4 (1.3)	2.0 (1.7)	*t*_93_ = 10.94	1.60, 2.31	< 0.0005

^a^*CME* Continuing Medical Education

^b^*SD =* Standard deviation

^c^*p* significance level

^d^Value index for awareness-related items: Disagree strongly = − 2, Disagree = − 1, Agree = 1, Agree strongly = 2

^e^Value for index for practice-related items: Never = 0, Rarely = 1, Half the time = 2, Usually = 3, Always = 4

^f^Likert scale: 0–10

### Factors affecting awareness, practice, confidence and knowledge

Face-to-face workshops were delivered within pre-defined time schedules. Online education was delivered according to the participant’s schedule. This created variation in the amount of time that elapsed between pre- and post-survey, based on the education delivery method. Therefore, logistic regression was only performed on pre- CME program awareness and practice.

Results of logistic regression of GP characteristics on pre- CME program awareness and practice are provided in Additional file 5: Table S4. The full logistic regression model for awareness was statistically significant *x*^2^ (12, *n* = 1205) = 48.58, *p* < 0.0005 and distinguished between respondents who were aware and those who were unaware of key dementia diagnosis and management topics prior to engaging in CME. Although the model correctly classified 78.3% of cases, it explained only 4% (Cox and Snell R-square) to 6.1% (Nagelkerke R-squared) of the variance in awareness and thus indicated that participant characteristics had little association with prior awareness of topic areas. The model for practice was significant *x*^2^ (12, *n* = 1204) = 58.69, *p* < 0.0005, correctly classified 53.3% of cases and explained 5 to 6.4% of the variance in practice.

At the pre- CME program stage, face-to-face participants were 2.15 times more likely than online participants to report awareness of key elements in the diagnosis and management of patients with dementia, and 1.5 times more likely to report that they incorporated recommended diagnosis and management actions in practice more often than not. GPs who had practised 11 to 15 years were twice as likely to endorse both awareness of topics and use of diagnosis and management methods in usual practice than were GPs with fewer than 5 years’ experience, controlling for other factors in the model. GPs with more than 20 years’ experience were similarly more likely to endorse actions in practice (*OR* = 1.84), than were GPs with fewer than 5 years’ experience.

Results of linear regression of GP characteristics on pre- CME program knowledge and confidence scores are provided in Additional file 6: Table S5. The total variance in self-reported pre- CME program knowledge scores explained by the model as a whole was 6.3% (Adjusted R Square), *F* (12, 1192) = 7.75, *p* < 0.0005. The total variance in confidence scores explained by the model as a whole was 8.6% (Adjusted R Square), *F* (12, 1191) = 10.45, *p* < 0.0005. Participants’ sex and years of practice experience contributed significantly to the model. Male participants had slightly higher self-rated pre- CME program knowledge and confidence scores compared with female participants. Having up to 5 years of practice experience was associated with lower self-rated knowledge and confidence scores than having more than 5 years’ practice experience, controlling for other variables.

Results of linear regression of GP characteristics on post-program knowledge and confidence scores are provided in Additional file 7: Table S6. The total variance in post-program knowledge scores explained by the model as a whole was 11.5%, *F* (12, 925) = 11.02, *p* < 0.0005 and for confidence was 14.9%, *F* (12, 925) = 14.50, *p* < 0.0005. Program delivery method, age and years of practice experience contributed significantly to the models for post-program knowledge and confidence scores. Face-to-face delivery was associated with slightly lower self-rated knowledge and confidence scores than online delivery. Compared to being less than 35 years of age and having fewer than five years practice experience (respectively), being at least 45 years of age and having between 5- and 20-years practice experience were associated with higher self-rated knowledge and confidence scores at post-program.

### Evaluation of the program and implementation

Over a quarter of GPs (*n* = 1005) returned a program evaluation survey. Most GPs (86.9%) felt that their learning needs were entirely met and that the program was entirely (91.9%) relevant to their practice. Examples of GP feedback are detailed in Additional file 8: Table S7. GPs noted that case studies and videos were impactful and suggested greater use of each to illustrate concepts. GPs requested more information on legal issues, assessing patient capacity, medication management and community services in poorly resourced regional and remote areas. Online participants requested concise, practical information over theory-driven content and readings. Face-to-face participants wanted local GPs and specialists to be the workshop facilitators and sought more locally relevant content.

Eight workshop facilitators (53.3%) completed a process evaluation survey. Examples of facilitator responses are detailed in Additional file 9: Table S8. The involvement of Primary Health Networks, the teaming of GPs and specialists as presenters, and the involvement of local professionals were seen as enablers of program delivery. Facilitators also encouraged greater use of case studies, more content regarding legal issues and local resources, and involving caregivers in workshops.

## Discussion

### Impact and outcome of GP education

GPs who participated in the survey evaluation of this accredited dementia-focused CME program indicated that their awareness, use of tools and management strategies in practice, and knowledge and confidence in diagnosing and managing dementia in practice, increased following participation in the CME. The program reached approximately 11% of the nation’s GPs during the study period. Participants were generally aware of most topics covered in the CME prior to participation in the program, but some GPs were unaware of key areas such as the difference between MCI and dementia, the cumulative anticholinergic effect of medications, and legal issues involved in care and management of dementia. Results for participants who were followed across all three time-points indicated that significant improvements were maintained with little decline at six to nine months post- CME program. The declining trend in follow-up survey highlights the importance of offering continuous training and keeping participants abreast of the most up-to-date knowledge and evidence. Outcomes of this evaluation informed tailoring of the educational material and delivery methods of the CME program (i.e. more information on legal issues; use of infographics, video content) as it continued to provide dementia-focused education through the triennium ending in 2019, and were a first step toward measuring impact.

### Factors affecting education outcomes

Online modules attracted the most professionals. Online education allowed GPs to proceed at their own pace and GPs completing modules online may have taken more time with the materials. Concurrently, results add to existing evidence that many GPs clearly remain interested in attending face-to-face training with colleagues [45, 48]. General Practitioners in regional, rural and remote areas were more likely than GPs working in urban areas to attend face-to-face workshops. Although consistent with previous CME literature noting the importance of interactive education and engagement with opinion leaders [48, 57], this outcome was both surprising and important in the context of increasing impetus to innovate through virtual / online applications in order to reach greater numbers at lower cost. Access to high-speed internet, as well as mobile phone service coverage, remains inconsistent in some rural, regional and remote areas in Australia and this has been noted as an issue in recent survey of rural doctors [58]. Engaging in the CME program online may have been impractical for some rural GPs and this may have influenced their choice of education delivery method in part.

Survey respondents who engaged in the face-to-face program included GPs who attended large GPCE and / or small group workshops. Professional conferences such as GPCE attract large numbers and are more suited to delivering short educational sessions and less-intensive CME programs. However, large numbers limit individualised interaction with participants and shorter sessions may allow less time for participants to absorb and reflect on materials. Small group workshops and/or online modules provide greater scope for delivering interactive, time-intensive programs. These differences may account in part for better outcomes for GPs who completed the program online.

GP characteristics had little practical association with awareness and current practice prior to the CME program and no association with their post-program outcomes. However, age and number of years in practice influenced self-reporting of knowledge and confidence. GPs with five or more years in practice reported more knowledge and confidence prior to the program than did their less experienced colleagues. GPs 45 years and older and those with between 5- and 20-years’ experience reported more knowledge and confidence after completing the CME than did their younger colleagues and those with fewer years in practice. Results are consistent with previous studies reporting that older, more experienced GPs feel more confident and knowledgeable than their younger counterparts regarding dementia-related care [35].

Six-hundred and fifty-seven GP registrars engaged in the program. Although all GPs reported better outcomes after participating in the CME program, registrars rated their own knowledge and confidence as below average going into workshops and just above average afterwards. Registrars having had less time in practice were less likely to have accumulated dementia-related case-knowledge compared with their more experienced colleagues. Registrars also participated in the shorter, overview workshop rather than the longer, 40-point CME workshop and this may have contributed in part to observed differences in age and experience effects. However, in a similar recent evaluation of dementia education, GP registrars self-reported pre-workshop levels of dementia knowledge were comparable to those of their more experienced supervisors [59].

Few GPs chose to continue research participation at six to nine months' follow-up. Participants were not asked about their motivation for participating. Age and practice experience appeared to influence longitudinal participation rates, as did CME program engagement method. Greater practice experience and possibly greater experience in dementia-related care may have made some GPs more receptive to the longer research commitment. GPs who interacted with colleagues face-to-face in group workshops may have been more willing to contribute to the CME research than were GPs who completed CME independently online.

### Participant and facilitator feedback on program delivery

Participant and facilitator feedback suggested that more tailored information on local services was desired. GPs emphasised the importance of involving local health care professionals who could provide local knowledge, as well as including caregivers of people living with dementia. As described previously [45, 53], CME program materials were reviewed by an expert steering committee including consumers and GPs and presented within a consistent framework in order to ensure program integrity. Fourteen local GPs and specialists completed train-the-trainer workshops and were engaged as CME workshop facilitators [45]. Workshop content was tailored for specific rural and remote regions in collaboration with these facilitators where possible. However, local GPs and specialists were not always available and tailored content was an exception.

Workshop facilitators noted geographical distance and small numbers of practising health professionals as barriers to conducting workshops in some regional and remote areas. Facilitators noted that some attendees in regional and remote venues had their first face-to-face professional contact with a geriatrician or specialist at the workshop. Australia’s population is concentrated primarily in major metropolitan areas along the country’s east and south-east coast and the south-west coast [60]. Lack of availability and/or access to professional colleagues with whom to collaborate in rural and remote areas are known barriers to referral for specialist services [44, 46, 47]. Although use of telehealth services is growing, high-speed internet access is limited in rural, regional and remote areas and rural GPs and non-GP specialists report this as a barrier to practice more broadly [58]. Primary Health Network collaboration plays an important role in small group workshop attendance, particularly in regional and remote areas. Face-to-face workshops on dementia provide invaluable, practical opportunities to connect GPs in regional and remote areas with local service providers, caregivers’ groups and national representatives. Communication with large national stakeholders and the local Primary Health Networks should commence as early as possible to overcome potential logistical challenges, to enable coordinated events, and to allow time for program promotion.

### Key areas identified for further training

Participants and facilitators flagged the need for more education addressing assessment of client capacity, legal issues and guidelines related to dementia and end of life issues, pharmacological management, and social impact for the client and family. These ongoing needs for training, education and specific guidelines to clinicians are widely acknowledged in the medical and legal community [38, 61]. Pharmacological management is similarly a significant and complex area in dementia care and management [62]. Future CME may be enhanced through devoting more time to presenting information on capacity-related issues and issues related to pharmacological management, in collaboration with specialists in these areas. Inclusion of people with dementia and caregivers, as well as representatives from consumer organisations, as session presenters may provide greater insight into the social impact of dementia for clients.

### Strengths and limitations

This study has several strengths. It is the first study to investigate the effectiveness of a nation-wide accredited dementia-focused CME program in Australia designed to enhance GP awareness, practice, knowledge and confidence in delivering timely diagnosis and management of dementia in general practice. Delivery and evaluation of the education program involved collaboration with multiple stakeholders including a national dementia advocacy organisation, a government-funded consortium of dementia education providers, private industry providers of medical education, and national medical professional accreditation organisations. The evaluation attracted a substantial, heterogeneous sample of GPs including a significant proportion of GPs practicing in regional areas.

The research ascertained GP self-appraisals of awareness, practice, knowledge and confidence regarding dementia-related topics. It synthesised data from self-evaluations, program evaluations, and process evaluations to identify current gaps in GP awareness, to track program effectiveness in improving self-reported capacity, and to identify areas for improving future training.

Limitations of this study include absence of a control group, use of a purpose-designed survey, participant self-selection, low survey response rates relative to the number of program participants, and use of self-report data. While participant self-selection could have introduced bias, the sample represented over 10% of Australian GPs. The study employed a one-group pretest-posttest design. It is possible that completing surveys prior to engaging in the education could have raised participants’ awareness of specific topics and therefore altered how they approached the education as well as their responses on the posttest.

Scales exist to measure GP dementia knowledge and awareness [59, 63, 64]. The aim of this study was to provide a tailored assessment of the efficacy of educational training provided to GPs in the first nation-wide CME dementia education program in Australia. In order to achieve the goals of this study, the survey was developed in alignment with the accredited CME program and did not utilise a psychometrically validated instrument.

The CME evaluation included self-report of current practice and intended practice, but it did not measure planned practice change [65–67] using theories such as commitment to practice change (CTC) [68] and it did not confirm longitudinal practice change beyond self-report [51]. While participation in interactive and multifaceted CME programs are beneficial for improving awareness and knowledge [69], it is one of many possible predictors of change in GPs’ self-reported practice and confidence. Unmeasured predictors such as GP attitudes, beliefs, values, personal experiences, peer influence and organisational factors may have influenced practice behaviour and self-report both pre- and post- study participation.

Low response rates are not uncommon in survey research with GPs and study response rates were similar to those reported elsewhere [63, 70, 71]. It is impossible to know with certainty the reasons for non-response amongst those GPs who did not engage in this CME, and amongst those GPs who took part in the CME but declined to participate in the survey. In Australia, CME point allocations are tracked in three-year training periods and most study data were obtained in the second and third year of the 2014–2016 training period [42]. GPs who had already accumulated sufficient CME points may have been less likely to enrol in the program. CME program evaluations were obtained only from GPs who applied for CME points. Those GPs who wished to participate longitudinally opted-in by providing an email address for follow-up. Thus, researchers were unable to follow GPs who did not provide contact information. We did not ask the respondents why they chose to engage in the dementia-focused CME modules, nor why they chose to complete any of the three evaluation questionnaires.

Survey participants self-selected and may have been more motivated than other GPs. Survey participants were generally younger than the national GP profile; GPs over 45 were under-represented. Follow-up response rates were low and comparison of GPs who submitted surveys at all three time-points with GPs who submitted surveys only at pre-, or at pre- and post- CME program indicated that longitudinal results primarily represented outcomes for face-to-face workshop attendees with greater practice experience. Longitudinal outcomes for online participants and GPs with less time in practice and registrars are not well represented. However, whilst acknowledging that there were differences between GPs who completed surveys at all three time-points and the majority who did not, results for completers and others were similar.

Although GP self-report provides insights into how GPs perceive their own abilities and educational needs, physician self-assessment is less accurate compared to external observations of competence [72]. Further research would be required to demonstrate whether the CME program narrowed the gap between presentation and diagnosis, improved accuracy of diagnosis or resulted in better management of care for persons with dementia and their families.

### Implications

Dementia-focused education is useful and relevant for GPs of varied experience levels. Most GPs had general awareness of dementia topics which improved further following education. Registrars had less knowledge and confidence in dementia-related assessment and management and may benefit from greater exposure to dementia education.

The CME program met GPs’ educational needs in both face-to-face and online formats. Time-poor GPs may prefer less background theory and more applied, practical information delivered in condensed formats such as reference sheets, webpages and video clips. The development of online learning modules provided a readily accessible free resource for GP self-directed CME training. The modules are now offered as an ongoing dementia training resource [73]. Online modules attracted larger numbers of GPs, but there is still a clear need for face-to-face workshops involving local health care professionals and particularly in regional and remote areas.

### Suggestions for future research

Meeting perceived educational needs and increases in self-rated indices of key outcomes may or may not translate to measurable improvements in practice. In order to address parameters influencing GP intention and implementation of practice change consistent with course objectives, future CME development and evaluation should involve a theory-based practice change approach with data triangulation to validate and enhance interpretation. In order to determine the sustained effectiveness of the CME program and identify emerging GP educational needs, future research should examine diagnosis rates and accuracy and adherence to clinical guidelines [28], gauge consumer satisfaction and the longer term effects on people living with dementia and their family caregivers, and identify variables associated with improved outcomes and sustained changes.

## Conclusions

GPs have a pivotal role in dementia recognition, assessment and referral, and are well-situated to provide continuing care co-ordination and support for patients. The scale of GP engagement showed that dementia CME programs, delivered both face-to-face and online, are relevant to GPs. The sustainability of self-reported change in key elements relating to dementia awareness, knowledge and confidence by health professionals taking part in this program showed that dementia CME programs may contribute to improving capacity to provide timely dementia diagnosis and management in general practice. Dementia-focused CME programs should be continued and updated as more research becomes available. Future research should measure outcomes in practice.

## Supplementary information

**Additional file 1.** Figure S1. Flowchart of survey response rates in a convenience sample of GPs engaged in dementia-focused CME.

**Additional file 2.** Table S1. Comparison of GP characteristics with national average.

**Additional file 3.** Table S2. Use of CME program delivery method by age and practice location (Major City, Regional, Remote).

**Additional file 4.** Table S3. Mean score change for subset of GPs who submitted surveys at each of two time-points.

**Additional file 5.** Table S4. Logistic regression of General Practitioner characteristics on pre-CME program awareness and practice.

**Additional file 6.** Table S5. Linear regression of General Practitioner characteristics on pre-CME program knowledge and confidence.

**Additional file 7.** Table S6. Linear regression of General Practitioner characteristics on post-CME program knowledge and confidence.

**Additional file 8.** Table S7. Exemplars of General Practitioner suggestions for improving future dementia-focused Continuing Medical Education programs.

**Additional file 9.** Table S8. Continuing Medical Education workshop facilitators’ responses to process evaluation survey questions.

**Additional file 10.** Completed SURGE (SUrvey Reporting GuidelinE) checklist.

## Data Availability

Restrictions apply to the availability of some data that support the findings of this study. These data were used with permission for the current study, and so are not publicly available. Data are however available from the authors upon reasonable request and with permission of Reed Medical Education.

## Abbreviations

ACRRM: Australian College of Rural and Remote Medicine
ARIA+: Accessibility and Remoteness Index of Australia
BPSD: Behavioural and Psychological Symptoms of Dementia
CME: Continuing Medical Education
GP: General Practitioner
GPCE: General Practice Conference and Exhibition
MCI: Mild Cognitive Impairment
RACGP: Royal Australian College of General Practitioners
