# Supplementary material for: GP awareness, practice, knowledge and confidence: evaluation of the first nation-wide dementia-focused continuing medical education program in Australia
Source: BMC Fam Pract. 2020 Jun 10;21:104. doi: 10.1186/s12875-020-01178-x (PMC7285709; doi:10.1186/s12875-020-01178-x)
Supplement: Supplementary file 1 — Additional file 1. Figure S1. Flowchart of survey response rates in a convenience sample of GPs engaged in dementia-focused CME. [file 12875_2020_1178_MOESM1_ESM.docx]

*N* = 3,923 unique GPs engaged in the dementia-focused Continuing Medical Education (CME) program

*n* = 2620 (66.8%) of GPs who engaged in the dementia-focused CME did not respond at baseline

*n* = 1303 (33.2%) of GPs who engaged in the dementia-focused CME completed a self-report survey of dementia-related awareness, practice, knowledge, and confidence at baseline

*n* = 1906 (48.6%) did not respond at post-program

*n* = 2017 (51.4%) completed a self-report survey at post-program

*n* = 138 (96.5%) did not respond at follow-up

*n* = 138 (3.5%) completed a self-report survey at follow-up
