# Supplementary material for: GP awareness, practice, knowledge and confidence: evaluation of the first nation-wide dementia-focused continuing medical education program in Australia
Source: BMC Fam Pract. 2020 Jun 10;21:104. doi: 10.1186/s12875-020-01178-x (PMC7285709; doi:10.1186/s12875-020-01178-x)
Supplement: Supplementary file 2 — Additional file 2. Table S1. Comparison of GP characteristics with national average. [file 12875_2020_1178_MOESM2_ESM.docx]

| Table S1. Comparison of GP characteristics with national average | | | |
| --- | --- | --- | --- |
| Variable | Participants (%) | National (%) | *p*-value^a^ |
| Female | 51.2 | 45.4 | *p* < .0005 |
| Registrars | 19.2 | 10.1 | *p* < .0005 |
| Age Groups |  |  |  |
| <35 | 24.9 | 14.1 | *p* < .0005 |
| 35-44 | 24.9 | 24.6 | *p* = .80 |
| 45- 54 | 20.7 | 24.3 | *p* = .003 |
| 55- 64 | 15.9 | 22.9 | *p* < .0005 |
| 65+ | 13.6 | 14.0 | *p* = .64 |
| Rurality^b^ |  |  |  |
| Major cities | 63.9 | 68.6 | *p* = .001 |
| Regional | 35.0 | 27.6 | *p* < .0005 |
| Remote | 1.1 | 3.8 | *p* < .0005 |
| ^a^ Significance set at *p* < 0.05.  ^b^ “Rurality” based on Accessibility / Remoteness Index of Australia (ARIA+) remoteness ratings. | | | |
