# Supplementary material for: GP awareness, practice, knowledge and confidence: evaluation of the first nation-wide dementia-focused continuing medical education program in Australia
Source: BMC Fam Pract. 2020 Jun 10;21:104. doi: 10.1186/s12875-020-01178-x (PMC7285709; doi:10.1186/s12875-020-01178-x)
Supplement: Supplementary file 3 — Additional file 3. Table S2. Use of CME program delivery method by age and practice location (Major City, Regional, Remote). [file 12875_2020_1178_MOESM3_ESM.docx]

| Table S2. Use of CME program delivery method by age and practice location (Major City, Regional, Remote) | | | | | | | | | | | | |
| --- | --- | --- | --- | --- | --- | --- | --- | --- | --- | --- | --- | --- |
| Program delivery method | General Practitioner age in years | | | | | | | Practice location rurality | | | | |
|  | < 35 | 35 to 44 | 45 to 54 | 55 to 64 | 65+ | *x^2^* | *p*^a^ (two-sided) | Major city | Regional | Remote | *x^2^* | *p* (two-sided) |
| Online,^b^ *n* (%) | 96 (16) | 191 (32) | 149 (25) | 93 (15) | 73 (12) |  |  | 435 (73) | 151 (25) | 7 (1) |  |  |
| Face-to-face,^c^ *n* (%) | 230 (32) | 136 (19) | 123 (17) | 115 (16) | 105 (15) |  |  | 365 (55) | 285 (43) | 8 (1) |  |  |
|  |  |  |  |  |  | 66.61 | < 0.0005 |  |  |  | 44.12 | < 0.0005 |

^a^*p* = significance level

^b^*n* = 1251

^c^*n* = 1311
