# Supplementary material for: GP awareness, practice, knowledge and confidence: evaluation of the first nation-wide dementia-focused continuing medical education program in Australia
Source: BMC Fam Pract. 2020 Jun 10;21:104. doi: 10.1186/s12875-020-01178-x (PMC7285709; doi:10.1186/s12875-020-01178-x)
Supplement: Supplementary file 4 — Additional file 4. Table S3. Mean score change for subset of GPs who submitted surveys at each of two time-points. [file 12875_2020_1178_MOESM4_ESM.docx]

| Table S3. Mean score change for subset of GPs who submitted surveys at each of two time-points | | | | | | |
| --- | --- | --- | --- | --- | --- | --- |
|  | Pre-education | Post-education | Difference between Pre-education and Post-education | Test statistic | 95% Confidence Interval | *p^b^* (two-sided) |
| Questions | Mean (*SD*)^a^ | Mean (*SD*) | Mean (*SD*) |  |  |  |
| Awareness^c^ (range -2 to 2) | | |  |  |  |  |
| GPs | 0.6 (0.7) | 1.5 (0.5) | 0.9 (0.8) | *t*_712_ = 32.37 | 0.86, 0.97 | < 0.0005 |
| GP registrars | 0.2 (0.6) | 1.1 (0.4) | 0.9 (0.6) | *t*_198_ = 21.67 | 0.82, 0.98 | < 0.0005 |
| Full subset | 0.5 (0.7) | 1.4 (0.5) | 0.9 (0.7) | *t*_911_ = 38.18 | 0.86, 0.96 | < 0.0005 |
| Practice^d^ (range 0 to 4) | |  |  |  |  |  |
| GPs | 2.2 (0.7) | 3.4 (0.5) | 1.1 (0.7) | *t*_709_ = 40.86 | 1.08, 1.19 | < 0.0005 |
| GP registrars | 1.6 (0.8) | 3.3 (0.5) | 1.7 (0.8) | *t*_198_ = 29.75 | 1.59, 1.82 | < 0.0005 |
| Full subset | 2.1 (0.8) | 3.3 (0.5) | 1.3 (0.8) | *t*_908_ = 48.00 | 1.21, 1.31 | < 0.0005 |
| Knowledge^e^ (range 0 to 10) | |  |  |  |  |  |
| GPs | 5.3 (1.7) | 7.5 (1.1) | 2.2 (1.5) | *t*_711_ = 38.09 | 2.08, 2.31 | < 0.0005 |
| GP registrars | 4.5 (1.6) | 6.4 (1.2) | 2.0 (1.3) | *t*_198_ = 21.58 | 1.79, 2.15 | < 0.0005 |
| Full subset | 5.1 (1.7) | 7.3 (1.2) | 2.1 (1.5) | *t*_910_ = 43.50 | 2.05, 2.25 | < 0.0005 |
| Confidence^e^ (range 0 to 10) | |  |  |  |  |  |
| GPs | 5.4 (1.8) | 7.6 (1.2) | 2.2 (1.6) | *t*_711_ = 37.36 | 2.08, 2.31 | < 0.0005 |
| GP registrars | 4.3 (1.7) | 6.2 (1.3) | 2.0 (1.3) | *t*_198_ = 20.83 | 1.77, 2.15 | < 0.0005 |
| Full subset | 5.2 (1.8) | 7.3 (1.3) | 2.1 (1.5) | *t*_910_ = 42.54 | 2.04, 2.24 | < 0.0005 |

^a^*SD* = Standard deviation

^b^*p* = significance level

^c^Value index for awareness-related items: Disagree strongly = – 2, Disagree = – 1, Agree = 1, Agree strongly = 2

^d^Value for index for practice-related items: Never = 0, Rarely = 1, Half the time = 2, Usually = 3, Always = 4

^e^Likert scale: 0 - 10
