# Supplementary material for: GP awareness, practice, knowledge and confidence: evaluation of the first nation-wide dementia-focused continuing medical education program in Australia
Source: BMC Fam Pract. 2020 Jun 10;21:104. doi: 10.1186/s12875-020-01178-x (PMC7285709; doi:10.1186/s12875-020-01178-x)
Supplement: Supplementary file 5 — Additional file 5. Table S4. Logistic regression of General Practitioner characteristics on pre-CME program awareness and practice. [file 12875_2020_1178_MOESM5_ESM.docx]

| Table S4. Logistic regression of General Practitioner characteristics on pre-CME program Awareness and Practice | | | | | | | |
| --- | --- | --- | --- | --- | --- | --- | --- |
|  | Awareness^a^  *n* = 1205 |  |  |  | Practice^b^  *n* = 1204 |  |  |
| Characteristic | Odds Ratio (95% CI^c^) | *S.E.*^d^ | *p*^e^ (two-sided) |  | Odds Ratio (95% CI) | *S.E.* | *p* (two-sided) |
| Male (reference female) | 1.12 (0.84 - 1.50) | 0.15 | 0.45 |  | 0.84 (0.65 - 1.05) | 0.12 | 0.15 |
| Years in practice |  |  |  |  |  |  |  |
| <5 (reference group) |  |  |  |  |  |  |  |
| 5 to 10 | 1.36 (0.89 - 2.07) | 0.22 | 0.15 |  | 1.34 (0.94 - 1.91) | 0.18 | 0.11 |
| 11 to 15 | 2.01 (1.07 - 3.77) | 0.32 | 0.03 |  | 2.22 (1.36 - 3.63) | 0.25 | 0.002 |
| 16 to 20 | 1.49 (0.74 – 2.98) | 0.36 | 0.26 |  | 1.18 (0.68 - 2.05) | 0.28 | 0.55 |
| >20 | 1.35 (0.72 - 2.54) | 0.32 | 0.35 |  | 1.84 (1.11 - 3.04) | 0.26 | 0.02 |
| Age |  |  |  |  |  |  |  |
| <35 (reference group) |  |  |  |  |  |  |  |
| 35 to 44 | 0.90 (0.58 - 1.38) | 0.22 | 0.63 |  | 1.17 (0.81 - 1.71) | 0.19 | 0.40 |
| 45 to 54 | 1.02 (0.57 - 1.83) | 0.30 | 0.94 |  | 1.32 (0.82 - 2.11) | 0.24 | 0.25 |
| 55 to 64 | 0.99 (0.50 - 1.99) | 0.35 | 0.99 |  | 1.56 (0.89 - 2.73) | 0.29 | 0.13 |
| 65+ | 1.65 (0.77- 3.55) | 0.39 | 0.20 |  | 1.47 (0.81 - 2.67) | 0.30 | 0.20 |
| Practice location |  |  |  |  |  |  |  |
| Major cities (reference group) |  |  |  |  |  |  |  |
| Regional | 1.13 (0.84 - 1.53) | 0.15 | 0.41 |  | 1.06 (0.82 - 1.36) | 0.13 | 0.67 |
| Remote | 3.86 (0.50 - 30.13) | 1.05 | 0.20 |  | 1.12 (0.38 – 3.34) | 0.56 | 0.84 |
| Face-to-face participants (ref. online participants) | 2.15 (1.58 – 2.93) | 0.16 | <0.0005 |  | 1.50 (1.17 - 1.92) | 0.13 | 0.001 |
| Constant | 1.78 | 0.16 | <0.0005 |  | 0.58 | 0.14 | <0.0005 |

CME, Continuing Medical Education

^a^Binary categories for awareness: Group 1, mean score of ≤ 0 in questions 1.1-1.15; Group 2, mean score > 0 in questions 1.1-1.15, average score of any individual item was between – 2 and 2

^b^Binary categories for practice: Group 1, mean score of ≤ 2 in total in questions 2.1-2.8; Group 2, score of > 2 in total in questions 2.1-2.8, average score of any individual item was between 0 and 4

^c^CI = Confidence Interval

^d^*S.E.* = Standard Error of Beta

^e^*p* = significance level; significant at ≤ 0.05
