# Supplementary material for: GP awareness, practice, knowledge and confidence: evaluation of the first nation-wide dementia-focused continuing medical education program in Australia
Source: BMC Fam Pract. 2020 Jun 10;21:104. doi: 10.1186/s12875-020-01178-x (PMC7285709; doi:10.1186/s12875-020-01178-x)
Supplement: Supplementary file 6 — Additional file 6. Table S5. Linear regression of General Practitioner characteristics on pre-CME program knowledge and confidence. [file 12875_2020_1178_MOESM6_ESM.docx]

| Table S5. Linear regression of General Practitioner characteristics on pre-CME program knowledge and confidence | | | | | | | | | | | | |
| --- | --- | --- | --- | --- | --- | --- | --- | --- | --- | --- | --- | --- |
|  | Knowledge, *n* = 1205 | | | | |  | Confidence, *n* = 1204 | | | | |  |
| Characteristic | *B*^a^ | *S.E. of B*^b^ | *β*^c^ | *p*^d^ (two-sided) | 95% CI^e^ |  | *B* | *S.E. of B* | *β* | *p* (two-sided) | 95% CI |  |
| Male (reference female) | 0.34 | 0.11 | 0.10 | 0.001 | 0.14, 0.55 |  | 0.42 | 0.11 | 0.12 | <0.0005 | 0.22, 0.63 |  |
| Years in practice |  |  |  |  |  |  |  |  |  |  |  |  |
| <5 (reference group) |  |  |  |  |  |  |  |  |  |  |  |  |
| 5 to 10 | 0.46 | 0.16 | 0.10 | 0.003 | 0.15, 0.77 |  | 0.55 | 0.16 | 0.12 | <0.0005 | 0.24, 0.86 |  |
| 11 to 15 | 0.71 | 0.21 | 0.12 | 0.001 | 0.29, 1.12 |  | 0.72 | 0.21 | 0.12 | 0.001 | 0.30, 1.14 |  |
| 16 to 20 | 0.60 | 0.24 | 0.09 | 0.01 | 0.12, 1.07 |  | 0.63 | 0.24 | 0.10 | 0.01 | 0.15, 1.10 |  |
| >20 | 0.79 | 0.22 | 0.21 | <0.0005 | 0.36, 1.22 |  | 0.84 | 0.22 | 0.22 | <0.0005 | 0.41, 1.28 |  |
| Age |  |  |  |  |  |  |  |  |  |  |  |  |
| <35 (reference group) |  |  |  |  |  |  |  |  |  |  |  |  |
| 35 to 44 | - 0.03 | 0.16 | -0.01 | 0.86 | - 0.35, 0.29 |  | 0.05 | 0.17 | 0.01 | 0.75 | - 0.27, 0.38 |  |
| 45 to 54 | 0.28 | 0.21 | 0.06 | 0.17 | - 0.12, 0.68 |  | 0.35 | 0.21 | 0.08 | 0.09 | - 0.06, 0.76 |  |
| 55 to 64 | 0.18 | 0.25 | 0.04 | 0.47 | - 0.30, 0.66 |  | 0.30 | 0.25 | 0.06 | 0.22 | - 0.18, 0.78 |  |
| 65+ | 0.21 | 0.26 | 0.04 | 0.43 | - 0.30, 0.71 |  | 0.43 | 0.26 | 0.08 | 0.10 | - 0.08, 0.94 |  |
| Practice location |  |  |  |  |  |  |  |  |  |  |  |  |
| Major cities (reference group) |  |  |  |  |  |  |  |  |  |  |  |  |
| Regional | 0.14 | 0.11 | 0.04 | 0.20 | - 0.07, 0.36 |  | 0.13 | 0.11 | 0.03 | 0.25 | - 0.09, 0.34 |  |
| Remote | - 0.06 | 0.47 | -0.003 | 0.91 | - 0.98, 0.87 |  | -0.21 | 0.47 | -0.01 | 0.65 | - 1.14, 0.72 |  |
| Face-to-face participants (ref. online participants) | - 0.07 | 0.11 | -0.02 | 0.52 | - 0.28, 0.14 |  | -0.03 | 0.11 | -0.01 | 0.77 | - 0.24, 0.18 |  |
| Constant | 4.42 | 0.21 |  | <0.0005 | 4.00, 4.84 |  | 4.21 |  |  | <0.0005 | 3.79, 4.63 |  |
| *R*^2^ |  |  |  |  |  | 0.07^f^ |  |  |  |  |  | 0.10^f^ |
| *F* |  |  |  |  |  | 7.75^f^ |  |  |  |  |  | 10.45^f^ |
| CME, Continuing Medical Education  ^a^*B* = unstandardized coefficient Beta  ^b^*S.E.* = Standard Error of Beta  ^c^*β* = standardized coefficient Beta  ^d^*p* = significance level; significant at ≤ 0.05  ^e^CI = Confidence Interval  ^f^*p* <0.0005, two-sided | | | | | | | | | | | | |
|  | | | | | | | | | | | | |
