# Supplementary material for: GP awareness, practice, knowledge and confidence: evaluation of the first nation-wide dementia-focused continuing medical education program in Australia
Source: BMC Fam Pract. 2020 Jun 10;21:104. doi: 10.1186/s12875-020-01178-x (PMC7285709; doi:10.1186/s12875-020-01178-x)
Supplement: Supplementary file 7 — Additional file 7. Table S6. Linear regression of General Practitioner characteristics on post-CME program knowledge and confidence. [file 12875_2020_1178_MOESM7_ESM.docx]

| Table S6. Linear regression of General Practitioner characteristics on post-CME program knowledge and confidence | | | | | | | | | | | | |
| --- | --- | --- | --- | --- | --- | --- | --- | --- | --- | --- | --- | --- |
|  | Knowledge, *n* = 926 | | | | |  | Confidence, *n* = 926 | | | | |  |
| Characteristic | *B*^a^ | *S.E. of B*^b^ | *β*^c^ | *p*^d^ (two-sided) | 95% CI^e^ |  | *B* | *S.E. of B* | *β* | *p* (two-sided) | 95% CI |  |
| Male (reference female) | 0.11 | 0.08 | 0.05 | 0.17 | - 0.05, 0.26 |  | 0.15 | 0.08 | 0.06 | 0.07 | - 0.01, 0.32 |  |
| Years in practice |  |  |  |  |  |  |  |  |  |  |  |  |
| <5 (reference group) |  |  |  |  |  |  |  |  |  |  |  |  |
| 5 to 10 | 0.42 | 0.12 | 0.14 | 0.001 | 0.19, 0.66 |  | 0.53 | 0.13 | 0.16 | <.0005 | 0.27, 0.78 |  |
| 11 to 15 | 0.40 | 0.16 | 0.10 | 0.01 | 0.08, 0.72 |  | 0.29 | 0.17 | 0.07 | 0.09 | - 0.05, 0.63 |  |
| 16 to 20 | 0.41 | 0.19 | 0.10 | 0.03 | 0.04, 0.78 |  | 0.39 | 0.20 | 0.09 | 0.05 | - 0.001, 0.79 |  |
| >20 | 0.24 | 0.17 | 0.10 | 0.17 | - 0.11, 0.58 |  | 0.25 | 0.19 | 0.09 | 0.17 | - 0.11, 0.62 |  |
| Age |  |  |  |  |  |  |  |  |  |  |  |  |
| <35 (reference group) |  |  |  |  |  |  |  |  |  |  |  |  |
| 35 to 44 | 0.08 | 0.13 | 0.03 | 0.55 | - 0.17, 0.32 |  | 0.14 | 0.13 | 0.04 | 0.30 | - 0.12, 0.40 |  |
| 45 to 54 | 0.53 | 0.16 | 0.18 | 0.001 | 0.21, 0.85 |  | 0.65 | 0.17 | 0.20 | <.0005 | 0.30, 0.99 |  |
| 55 to 64 | 0.43 | 0.19 | 0.13 | 0.03 | 0.05, 0.80 |  | 0.59 | 0.20 | 0.17 | 0.004 | 0.19, 0.98 |  |
| 65+ | 0.60 | 0.20 | 0.18 | 0.002 | 0.21, 1.00 |  | 0.82 | 0.21 | 0.22 | <.0005 | 0.41, 1.24 |  |
| Practice location |  |  |  |  |  |  |  |  |  |  |  |  |
| Major cities (reference group) |  |  |  |  |  |  |  |  |  |  |  |  |
| Regional | - 0.07 | 0.08 | - 0.03 | 0.39 | - 0.23, 0.09 |  | - 0.03 | 0.09 | - 0.01 | 0.71 | - 0.20, 0.14 |  |
| Remote | 0.51 | 0.36 | 0.04 | 0.16 | - 0.20, 1.22 |  | 0.31 | 0.39 | 0.02 | 0.42 | - 0.45, 1.07 |  |
| Face-to-face participants (ref. online participants) | - 0.39 | 0.08 | - 0.16 | <0.0005 | - 0.55, - 0.23 |  | - 0.53 | 0.09 | - 0.19 | <0.0005 | - 0.70, - 0.35 |  |
| Constant | 7.38 | 0.17 |  | <0.0005 | 7.05, 7.72 |  | 7.47 |  |  | <0.0005 | 7.11, 7.82 |  |
| *R*^2^ |  |  |  |  |  | 0.13^f^ |  |  |  |  |  | 0.16^f^ |
| *F* |  |  |  |  |  | 11.02^f^ |  |  |  |  |  | 14.50^f^ |
| CME, Continuing Medical Education  ^a^B = unstandardized coefficient Beta  ^b^S.E. = Standard Error of Beta  ^c^*β* = standardized coefficient Beta  ^d^*p* = significance level; significant at ≤ 0.05  ^e^95% CI = 95% Confidence Interval  *p* < 0.0005, two-sided | | | | | | | | | | | | |
