# Supplementary material for: GP awareness, practice, knowledge and confidence: evaluation of the first nation-wide dementia-focused continuing medical education program in Australia
Source: BMC Fam Pract. 2020 Jun 10;21:104. doi: 10.1186/s12875-020-01178-x (PMC7285709; doi:10.1186/s12875-020-01178-x)
Supplement: Supplementary file 8 — Additional file 8. Table S7. Exemplars of General Practitioner suggestions for improving future dementia-focused Continuing Medical Education programs. [file 12875_2020_1178_MOESM8_ESM.docx]

| Table S7. Exemplars of General Practitioner suggestions for improving future dementia-focused Continuing Medical Education | |
| --- | --- |
| Main themes | Example suggestions |
| Capacity | “A bit more time on assessing capacity vs cognitive impairment.” |
| Driving | “Increased focus on driving. In particular more detail on what the National and State authorities recommend regarding dementia and driving.” |
| End of life issues | “Perhaps more focus on end of life issues in dementia.” |
| Legal issues | “More detail on legal issues especially re: families.” |
| Impact on people | “The topic of dementia and impact on people’s lives is huge. The practical application for me is really where the rubber meets the road.” |
| Social services | “Some education on emergency social support for people with dementia who present with acute problem.” |
| Prevention | “More emphasis on prevention and the role of brain training.” |
| Medications | “A little more knowledge about the suitable pharmatherapy to manage dementia, latest drugs, and new additions could be beneficial.” |
| Care team | “Perhaps a little more focus on establishing "the team" who will help care for the patient with dementia and their family…The role of the specialist and "the clinic" was alluded to, however how those roles overlap or what is "ideal" is so location dependent it would be good to look at "models of team care" that can be adapted.” |
| Practice in rural areas | “Was really effective but discussion of lack of services in more rural areas and how to cope with this would be helpful.” |
| Practical handouts | “Handouts for Dementia Services phone numbers and Advanced Care Directive.” |
| Local knowledge | “Involve more local health care professionals.” |
| Local services | “[More] local resources and access pathways for coordination.” |
| Specialists | “Provide more interviews with specialist.” |
| Nurses and allied health | “Allied Health and RACF [Residential Aged Care Facility] nursing staff also to present.” |
| Inclusivity | “Include [caregivers] of patients with cognitive impairment.” |
| Break into smaller groups | “Probably more - interaction - e.g. having a case and getting into groups - to find ways to manage dementia in the community- and presenting it at the end.” |
| Case studies | “More stories of successful and challenging cases and how they progressed.” |
| Videos of patients’ stories | “The video clips of the stories of the patients were very touching and gave a clear idea of how dementia affects the families and the community.” |
| Video tutorials | “Video tutorials were very helpful. Easier absorbed information than textual knowledge.” |
| Humour | “If anyone can add humour to the subject that would improve attention and focusing on the topic.” |
| Pre-conference notes | “Dementia and Alzheimer is big topic to cover even with 6 hours of [CME]… pre-conference notes will be helpful as many of the information/concept are new to me.” |
| Condensed information | “More diagrams/flowcharts.” |
