# Supplementary material for: GP awareness, practice, knowledge and confidence: evaluation of the first nation-wide dementia-focused continuing medical education program in Australia
Source: BMC Fam Pract. 2020 Jun 10;21:104. doi: 10.1186/s12875-020-01178-x (PMC7285709; doi:10.1186/s12875-020-01178-x)
Supplement: Supplementary file 9 — Additional file 9. Table S8. Continuing Medical Education workshop facilitators’ responses to process evaluation survey questions. [file 12875_2020_1178_MOESM9_ESM.docx]

| Table S8. Continuing Medical Education workshop facilitators’ responses to process evaluation survey questions | | | |
| --- | --- | --- | --- |
| Questions | Key topic | Main themes | Example responses |
| What barriers and/or enablers did you experience delivering the Continuing Medical Education (CME) sessions? | | | |
|  | Barriers | Turnout | “From an organisational standpoint, getting people to come along, getting help from PHNs [Primary Health Networks], getting people to fit things into their schedules….” |
|  |  | Weekend workshops | “Difficult to get GPs to attend events on a Saturday. Plus GPs have weekend’s on-call commitment.” |
|  |  | Geography/ location | “I presented in [multiple regional locations]. Transportation can be a bit difficult there...” |
|  |  | Topic area | “Many GPs don't have patients in their practice with dementia, or ones they know have dementia…So there may not be the motivation to come to [CME] on dementia as there would be for other topics.” |
|  | Enablers | Weekend workshops | “The workshops are on weekends. All accommodation, travel etc. is already organized [for facilitators].” |
|  |  | Primary Health Networks (PHN) | “…once the PHNs were well established, a very good relationship was established and effective promotion and cooperation in supporting the meetings occurred nationally.” |
|  |  | Planning and organisation | “With the way things were set-up in advance there was sufficient time to organise timing…The organisation was an enabler.” |
|  |  | Content | “The content was pitched at the right level, it didn't leave many people behind.” |
|  |  | Local knowledge | “…understanding of local resources/limitations assists in delivering more tailored information.” |
| What do you feel were the reasons behind these barriers and enablers? | | | |
|  | Reasons for barriers | Geography/ location | “I guess you could say geography is a barrier for parts of rural and regional or more remote areas because there aren't as many practicing professionals in some areas.” |
|  |  | Topic area | “GPs are very stretched for time and everyone wants them to be trained in their [topic] area, so they can be selective.” |
|  |  | Timing within 3-year CME training period | “Most GPs have their points by the 1st or 2nd year, so it wasn't as popular [in the 3rd year].” |
|  |  | Weekend workshops | “As session was an all day Saturday program--difficult for GPs to attend, especially in regional areas.” |
|  | Reasons for enablers | Project organisation | “The program’s ability to organize and pay for accommodation, travel etc. allows participation without the hassle of organizing this yourself.” |
|  |  | Presence of specialists | “A good thing was that experienced facilitators were there to discuss cases at a higher level. For many people attending, it may have been their first opportunity to speak with a specialist about cases.” |
|  |  | Case study approach | “The case studies worked really well, in fact I'd say they should be a more prominent part of the full session.” |
|  |  | Primary Health Network | “Working through the PHNs was useful, a good way of promoting the training.” |
|  |  | Small group size | “Twenty people was a good number for sessions, good for discussions. Practice Nurses joined in and talked about their side of things.” |
| Do you have any comments regarding your preparation for presenting the CME? | | | |
|  | Enablers | Communication with other facilitators | “[Facilitator name] is excellent at keeping me up to date with any new slides, information etc. and sends through updated information. There is opportunity for meeting up before the sessions…to talk about the place we are presenting at, the expected participants and their backgrounds e.g. GP’s, nursing, allied health…We talk about new developments in research or new examples we can give that are relevant.” |
|  |  | Prepared materials | “I've never been able to give a talk that was already written, but these were so comprehensive and updated and it wasn't a problem.” |
|  |  | Tailoring and targeting engagement | “For example, the 1st session I go over how to use the GP COG [screening tool]. I get volunteers to [roleplay] the [caregiver] and we go through it together. Doing it live holds their attention…Some GPs need every little detail, some need overall approaches to apply to the problem.” |
| Do you have any comments or suggestions regarding the organization of the training program? | | | |
|  | Comments | Primary Health Networks | “[Organiser] uses the PHN to promote the program. That works very well. I mean sometimes the turnout's a bit disappointing. For instance in [regional location], but of course there are less GPs there. But it's still very important to go to the regional areas.” |
|  |  | Facilities and Resources | “The locations are always comfortable and well catered. This helps participants feel comfortable and allows for an optimal learning environment. Equipment is working and help on hand for any technology difficulties. Surveys etc. are printed off and available for participants on the day.” |
|  | Suggestions | Need to improve event promotion | “Some PHNs seem to be more in contact with their GPs than others. Like with [regional location], it didn't go through because they didn't seem to be able to get the word out there and no one signed up. To get 20 [people] to turn up is good.” |
|  |  | Organise environments to promote discussion | “[Re setup] We found the U-shaped table set-up worked best…I prefer the closed environment where people have to talk to one another.” |
| What would you do differently if you were to present future sessions? (i.e. aims and outcomes, content, presentation methods, timing, follow-up and sustainability)? | | | |
|  | Barriers | Fewer or shorter sessions needed | “The [CME] was probably a bit long…It's a full day and people fade…If you leave room at the end of the session for questions, it goes down a bit better…People are shattered if you stay to finish at 4.30 on a Saturday.” |
|  | Enablers | More time/ discussion about legal issues | “…more about the legal stuff, which is what most GPs struggle with--the legal-ese end. People have no idea and they're asked about enduring guardianship, etc.….it’s all very complex and people just have no idea.” |
|  |  | Local knowledge/ local content | “At [location] we presented a session on the needs of Aboriginal and Torres Strait Islander Communities, and that was good. I'd say it's good to have a bit of local content wherever you are doing it.” |
|  |  | Interactive case scenarios | “…people learn best if they're actually active in the process…it sticks better if you get people to think about how they would manage a patient and integrate that knowledge into their practice.” |
|  |  | Greater inclusiveness | “Include more GP Trainees, GP Practice Nurses, and Allied Health Professionals.” |
| Do you have any additional comments/suggestions? | | | |
|  |  | Value of GP and specialist team | “…there is great strength in having a GP and a geriatrician in the room. Having the validity of another GP saying ‘Yeah, that's how you do it’ was so helpful. Sometime when you go to these things there is an edge between the secondary and primary teams. Having the GP and geriatrician together minimized this...” |
