# Supplementary material for: GP awareness, practice, knowledge and confidence: evaluation of the first nation-wide dementia-focused continuing medical education program in Australia
Source: BMC Fam Pract. 2020 Jun 10;21:104. doi: 10.1186/s12875-020-01178-x (PMC7285709; doi:10.1186/s12875-020-01178-x)
Supplement: Supplementary file 10 — Additional file 10. Completed SURGE (SUrvey Reporting GuidelinE) checklist. [file 12875_2020_1178_MOESM10_ESM.docx]

| **SURGE (SUrvey Reporting GuidelinE) checklist** | | |
| --- | --- | --- |
| **Reporting Item** |  | Reported on page # |
| **Title and Abstract** |  |  |
| Design of study stated | Yes | 3 |
| **Introduction** | | |
| Explanation of need for research in the context of previous work in relevant fields | Yes | 5 - 7 |
| Purpose/aim of paper explicitly stated | Yes | 8 |
| **Methods** | | |
| **Research Tool** | | |
| Description of the questionnaire | Yes | 9 - 10 |
| Core questions provided | Yes | 14 (Table 2) |
| Existing tool, psychometric properties presented | N/A | N/A |
| Existing tool, references to original work provided | N/A | N/A |
| New tool, procedures to develop and pre-test provided | Yes | 9 |
| New tool, reliability and validity reported | No | N/A |
| Description of the scoring procedures provided | Yes | 9 |
| **Sample Selection** |  |  |
| Description of survey population and sample | Yes | 8, 12 |
| Description of representativeness of the sample | Yes | 8, 12 |
| Sample size calculation or rationale/justification presented | No | N/A |
| **Survey Administration** |  |  |
| Mode of administration | Yes | 8, 9 |
| Information on the type and number of contacts provided | Yes | 8 - 10 |
| Information on financial or other incentives provided | Yes | 7, 9 |
| Description of who approached potential participants | Yes | 8 - 10 |
| **Analysis** |  |  |
| Method of data analysis described | Yes | 11 - 12 |
| Method for analysis of nonresponse error provided | No | N/A |
| Method for calculating response rate provided | Yes | 11 |
| Definitions for complete versus partial completions provided | Yes | 11 |
| Methods for handling item missing data provided | Yes | 11 |
| **Results** |  |  |
| Response rate reported | Yes | 13 |
| All respondents accounted for | Yes | 13 (Additional File 1: Figure S1) |
| Information on how non-respondents differ from respondents | Yes | 13 (Additional File 2: Table S1); 15 (Table 3) |
| Results clearly presented | Yes | 12 - 17 |
| Results address objectives | Yes | 12 - 17 |
| **Discussion** |  |  |
| Results summarized referencing study objectives | Yes | 18 – 22, 25 - 26 |
| Strengths of the study stated | Yes | 22 - 23 |
| Limitations of the study stated | Yes | 23 - 25 |
| Generalizability of results discussed | Yes | 23 - 25 |
| **Ethical Quality Indicators** |  |  |
| Study funding reported | Yes | 7, 27 |
| Research Ethics Board (REB) review reported | Yes | 8, 26 |
| Subject consent procedures reported | Yes | 8, 26 - 27 |
| Bennett C, Khangura S, Brehaut JC, Graham ID, Moher D, Potter BK, et al. Reporting guidelines for survey research: an analysis of published guidance and reporting practices. PLoS Med. 2011;8(8):e1001069. https://doi.org/10.1371/journal.pmed.1001069 | | |
